# 1-(5-Hydroxy-7-methoxy-2,2-dimethyl-2*H*-chromen-6-yl)ethan-1-one

**DOI:** 10.1107/S1600536810018374

**Published:** 2010-05-22

**Authors:** Bingjing Liu, Guangying Chen, Changchun Cen, Xinming Song, Changri Han

**Affiliations:** a Key Laboratory of Tropical Medicinal Plant Chemistry of the Ministry of Education, Hainan Normal University, College of Chemistry & Chemical Engineering, Hainan Normal University, Haikou 571158, People’s Republic of China

## Abstract

The title chromene, C_14_H_16_O_4_, was isolated from the stems of *Polyalthia plagioneura* Diels. The mol­ecular structure is stabilized by an intra­molecular O–H⋯O hydrogen bond, which generates an *S*(6) ring. In the crystal, the mol­ecules are linked by C—H⋯O inter­actions, generating [010] chains.

## Related literature

For medicinal and botanical background to the title compound, see: Allan *et al.* (1969 ▶); Manandhar *et al.* (1985 ▶); Li *et al.* (1997 ▶).
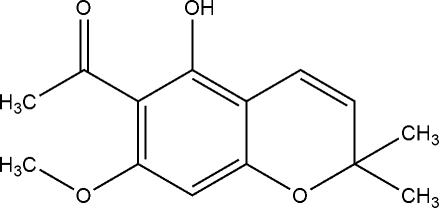


## Experimental

### 

#### Crystal data

C_14_H_16_O_4_
*M* *_r_* = 248.28
Triclinic, 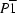

*a* = 7.3797 (9) Å
*b* = 8.0066 (10) Å
*c* = 11.2878 (14) Å
α = 77.948 (1)°
β = 77.411 (1)°
γ = 84.465 (2)°
*V* = 635.67 (14) Å^3^
*Z* = 2
Mo *K*α radiation
μ = 0.10 mm^−1^
*T* = 298 K
0.45 × 0.40 × 0.39 mm

#### Data collection

Bruker SMART CCD diffractometer
Absorption correction: multi-scan (*SADABS*; Bruker, 1997 ▶) *T*_min_ = 0.959, *T*_max_ = 0.964
3335 measured reflections
2217 independent reflections
1361 reflections with *I* > 2σ(*I*)
*R*_int_ = 0.019

#### Refinement

*R*[*F* ^2^ > 2σ(*F* ^2^)] = 0.059
*wR*(*F* ^2^) = 0.159
*S* = 1.08
2217 reflections
167 parameters
H-atom parameters constrained
Δρ_max_ = 0.26 e Å^−3^
Δρ_min_ = −0.23 e Å^−3^

### 

Data collection: *SMART* (Bruker, 1997 ▶); cell refinement: *SAINT* (Bruker, 1997 ▶); data reduction: *SAINT*; program(s) used to solve structure: *SHELXS97* (Sheldrick, 2008 ▶); program(s) used to refine structure: *SHELXL97* (Sheldrick, 2008 ▶); molecular graphics: *SHELXTL* (Sheldrick, 2008 ▶); software used to prepare material for publication: *SHELXTL*.

## Supplementary Material

Crystal structure: contains datablocks global, I. DOI: 10.1107/S1600536810018374/hb5429sup1.cif

Structure factors: contains datablocks I. DOI: 10.1107/S1600536810018374/hb5429Isup2.hkl

Additional supplementary materials:  crystallographic information; 3D view; checkCIF report

## Figures and Tables

**Table 1:** Hydrogen-bond geometry (Å, °)

*D*—H⋯*A*	*D*—H	H⋯*A*	*D*⋯*A*	*D*—H⋯*A*
O2—H2⋯O3	0.82	1.75	2.479 (2)	147
C8—H8⋯O3^i^	0.93	2.48	3.374 (3)	162

Symmetry code: (i) 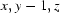.

**Fig. 1.**
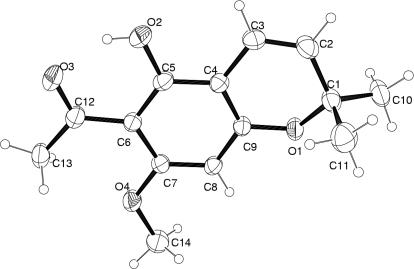
View of the title compound with displacement ellipsoids drawn at the 30% probability level.

## supplementary crystallographic information

### Comment

The title chromene was isolated from plants such as *Remirea maritima* (Allan *et al.*, 1969), *Euodia lunu-ankenda* (Manandhar *et al.*, 1985) and *Evodia lepta* (Li *et al.*, 1997). In our ongoing studies of natural products with biological activity we isolated the chromene from the 75% EtOH extract of the stems of *Polyalthia plagioneura*, a plant used as a flok medicine which were collected from Bawangling, Hainan Province, P. R. China. We have undertaken the X-ray crystal structure analysis of the title compound in order to establish its molecular structure and relative stereochemistry.

The hydrogen bonds and angles are listed in Table 1.

### Experimental

Air-dried stems of *Polyalthia plagioneura* (20 kg) were ground and percolated (4 × 3 h) with 75% EtOH at 60°C, which was suspended in 5 L water and then partitioned with chloroform, ethyl acetate and n-BuOH, successively, yielding a chloroform extract, an ethyl acetate extract and a n-BuOH extract, respectively. The chloroform extract was subjected to a silica gel CC column using petroleum ether as first eluent and then increasing the polarity with EtOAc, to afford 33 fractions. Fraction 5 was further separated by column chromatography with a gradient of chloroform–ether–EtOAc to give the title compound. The crude product was recrystallised from ethyl acetate to yield colourless blocks of (I).

### Refinement

H atoms bonded to C atoms were palced in geometrically calculated position and were refined using a riding model, with *U*_iso_(H) = 1.2 Ueq(C). H atoms attached to O atoms were found in a difference Fourier synthesis and were refined using a riding model, with the O—H distances fixed as initially found and with *U*_iso_(H) values set at 1.5 *U*eq(O).

### Crystal data

**Table:**

C_14_H_16_O_4_	*Z* = 2
*M**_r_* = 248.28	*F*(000) = 264
Triclinic, *P*1	*D*_x_ = 1.297 Mg m^−^^3^
Hall symbol: -P 1	Mo *K*α radiation, λ = 0.71073 Å
*a* = 7.3797 (9) Å	Cell parameters from 1208 reflections
*b* = 8.0066 (10) Å	θ = 2.6–24.1°
*c* = 11.2878 (14) Å	µ = 0.10 mm^−^^1^
α = 77.948 (1)°	*T* = 298 K
β = 77.411 (1)°	Block, colourless
γ = 84.465 (2)°	0.45 × 0.40 × 0.39 mm
*V* = 635.67 (14) Å^3^	

### Data collection

**Table:**

Bruker SMART CCD diffractometer	2217 independent reflections
Radiation source: fine-focus sealed tube	1361 reflections with *I* > 2σ(*I*)
graphite	*R*_int_ = 0.019
ω scans	θ_max_ = 25.0°, θ_min_ = 1.9°
Absorption correction: multi-scan (*SADABS*; Bruker, 1997)	*h* = −8→8
*T*_min_ = 0.959, *T*_max_ = 0.964	*k* = −8→9
3335 measured reflections	*l* = −13→13

### Refinement

**Table:**

Refinement on *F*^2^	Primary atom site location: structure-invariant direct methods
Least-squares matrix: full	Secondary atom site location: difference Fourier map
*R*[*F*^2^ > 2σ(*F*^2^)] = 0.059	Hydrogen site location: inferred from neighbouring sites
*wR*(*F*^2^) = 0.159	H-atom parameters constrained
*S* = 1.08	*w* = 1/[σ^2^(*F*_o_^2^) + (0.0801*P*)^2^] where *P* = (*F*_o_^2^ + 2*F*_c_^2^)/3
2217 reflections	(Δ/σ)_max_ < 0.001
167 parameters	Δρ_max_ = 0.26 e Å^−^^3^
0 restraints	Δρ_min_ = −0.23 e Å^−^^3^

### Special details

**Table:**

Geometry. All esds (except the esd in the dihedral angle between two l.s. planes) are estimated using the full covariance matrix. The cell esds are taken into account individually in the estimation of esds in distances, angles and torsion angles; correlations between esds in cell parameters are only used when they are defined by crystal symmetry. An approximate (isotropic) treatment of cell esds is used for estimating esds involving l.s. planes.
Refinement. Refinement of *F*^2^ against ALL reflections. The weighted *R*-factor wR and goodness of fit *S* are based on *F*^2^, conventional *R*-factors *R* are based on *F*, with *F* set to zero for negative *F*^2^. The threshold expression of *F*^2^ > σ(*F*^2^) is used only for calculating *R*-factors(gt) etc. and is not relevant to the choice of reflections for refinement. *R*-factors based on *F*^2^ are statistically about twice as large as those based on *F*, and *R*- factors based on ALL data will be even larger.

### Fractional atomic coordinates and isotropic or equivalent isotropic displacement parameters (Å^2^)

**Table:**

	*x*	*y*	*z*	*U*_iso_*/*U*_eq_	
O1	0.1836 (2)	0.20248 (18)	0.25325 (13)	0.0529 (5)	
O2	0.2390 (2)	0.76852 (19)	0.29434 (15)	0.0659 (5)	
H2	0.2508	0.8241	0.3453	0.099*	
O3	0.2742 (3)	0.8220 (2)	0.49648 (17)	0.0720 (6)	
O4	0.2615 (2)	0.30717 (19)	0.63521 (13)	0.0604 (5)	
C1	0.2377 (3)	0.2453 (3)	0.11859 (19)	0.0534 (7)	
C2	0.2004 (4)	0.4317 (3)	0.0732 (2)	0.0584 (7)	
H2A	0.1813	0.4684	−0.0072	0.070*	
C3	0.1938 (3)	0.5467 (3)	0.1434 (2)	0.0546 (7)	
H3	0.1751	0.6624	0.1116	0.066*	
C4	0.2160 (3)	0.4900 (3)	0.27044 (19)	0.0431 (6)	
C5	0.2349 (3)	0.6020 (3)	0.3466 (2)	0.0451 (6)	
C6	0.2498 (3)	0.5431 (3)	0.47159 (19)	0.0419 (5)	
C7	0.2432 (3)	0.3632 (3)	0.51639 (19)	0.0429 (6)	
C8	0.2232 (3)	0.2528 (3)	0.44272 (19)	0.0433 (5)	
H8	0.2184	0.1360	0.4741	0.052*	
C9	0.2101 (3)	0.3175 (3)	0.32112 (19)	0.0411 (5)	
C10	0.1212 (4)	0.1353 (3)	0.0740 (2)	0.0695 (8)	
H10A	0.1443	0.0172	0.1090	0.104*	
H10B	0.1534	0.1517	−0.0146	0.104*	
H10C	−0.0081	0.1672	0.0992	0.104*	
C11	0.4434 (4)	0.1947 (4)	0.0849 (2)	0.0791 (8)	
H11A	0.5131	0.2610	0.1191	0.119*	
H11B	0.4831	0.2157	−0.0035	0.119*	
H11C	0.4640	0.0754	0.1176	0.119*	
C12	0.2686 (3)	0.6685 (3)	0.5448 (2)	0.0501 (6)	
C13	0.2816 (3)	0.6241 (3)	0.6777 (2)	0.0598 (7)	
H13A	0.2903	0.7267	0.7070	0.090*	
H13B	0.3900	0.5500	0.6867	0.090*	
H13C	0.1727	0.5670	0.7251	0.090*	
C14	0.2647 (4)	0.1275 (3)	0.6819 (2)	0.0717 (8)	
H14A	0.1481	0.0845	0.6811	0.108*	
H14B	0.2846	0.1051	0.7652	0.108*	
H14C	0.3635	0.0720	0.6310	0.108*	

### Atomic displacement parameters (Å^2^)

**Table:**

	*U*^11^	*U*^22^	*U*^33^	*U*^12^	*U*^13^	*U*^23^
O1	0.0793 (12)	0.0426 (10)	0.0413 (9)	−0.0099 (8)	−0.0147 (8)	−0.0124 (7)
O2	0.0982 (14)	0.0346 (10)	0.0677 (11)	−0.0076 (8)	−0.0267 (10)	−0.0039 (8)
O3	0.1000 (15)	0.0432 (11)	0.0818 (13)	−0.0087 (9)	−0.0265 (10)	−0.0213 (9)
O4	0.0955 (14)	0.0452 (10)	0.0444 (9)	−0.0021 (8)	−0.0218 (8)	−0.0104 (8)
C1	0.0680 (18)	0.0559 (16)	0.0383 (12)	−0.0089 (12)	−0.0100 (11)	−0.0119 (11)
C2	0.0750 (18)	0.0597 (17)	0.0403 (13)	−0.0108 (13)	−0.0151 (12)	−0.0023 (12)
C3	0.0694 (17)	0.0430 (14)	0.0500 (14)	−0.0079 (11)	−0.0164 (12)	0.0010 (11)
C4	0.0455 (14)	0.0384 (13)	0.0461 (13)	−0.0026 (10)	−0.0111 (10)	−0.0077 (10)
C5	0.0464 (14)	0.0335 (12)	0.0547 (14)	−0.0029 (9)	−0.0110 (10)	−0.0059 (10)
C6	0.0422 (13)	0.0392 (13)	0.0467 (13)	−0.0003 (10)	−0.0109 (10)	−0.0126 (10)
C7	0.0487 (14)	0.0425 (13)	0.0374 (12)	−0.0004 (10)	−0.0089 (10)	−0.0087 (10)
C8	0.0545 (15)	0.0321 (12)	0.0432 (12)	−0.0029 (10)	−0.0097 (10)	−0.0069 (10)
C9	0.0452 (14)	0.0394 (13)	0.0410 (12)	−0.0041 (10)	−0.0089 (10)	−0.0114 (10)
C10	0.097 (2)	0.0722 (19)	0.0490 (14)	−0.0204 (15)	−0.0228 (14)	−0.0173 (13)
C11	0.076 (2)	0.092 (2)	0.0690 (18)	−0.0014 (15)	−0.0054 (15)	−0.0255 (16)
C12	0.0460 (14)	0.0452 (15)	0.0628 (15)	−0.0024 (11)	−0.0109 (11)	−0.0191 (12)
C13	0.0643 (17)	0.0611 (17)	0.0636 (16)	−0.0029 (12)	−0.0148 (12)	−0.0322 (13)
C14	0.113 (2)	0.0547 (17)	0.0450 (14)	−0.0050 (15)	−0.0215 (14)	0.0016 (12)

### Geometric parameters (Å, °)

**Table:**

O1—C9	1.367 (2)	C6—C12	1.460 (3)
O1—C1	1.460 (2)	C7—C8	1.371 (3)
O2—C5	1.341 (2)	C8—C9	1.383 (3)
O2—H2	0.8200	C8—H8	0.9300
O3—C12	1.237 (3)	C10—H10A	0.9600
O4—C7	1.354 (2)	C10—H10B	0.9600
O4—C14	1.424 (3)	C10—H10C	0.9600
C1—C2	1.493 (3)	C11—H11A	0.9600
C1—C10	1.513 (3)	C11—H11B	0.9600
C1—C11	1.519 (3)	C11—H11C	0.9600
C2—C3	1.326 (3)	C12—C13	1.490 (3)
C2—H2A	0.9300	C13—H13A	0.9600
C3—C4	1.451 (3)	C13—H13B	0.9600
C3—H3	0.9300	C13—H13C	0.9600
C4—C9	1.381 (3)	C14—H14A	0.9600
C4—C5	1.401 (3)	C14—H14B	0.9600
C5—C6	1.415 (3)	C14—H14C	0.9600
C6—C7	1.426 (3)		
			
C9—O1—C1	118.89 (16)	O1—C9—C4	120.78 (18)
C5—O2—H2	109.5	O1—C9—C8	116.67 (18)
C7—O4—C14	118.00 (17)	C4—C9—C8	122.51 (19)
O1—C1—C2	110.59 (18)	C1—C10—H10A	109.5
O1—C1—C10	104.22 (17)	C1—C10—H10B	109.5
C2—C1—C10	112.3 (2)	H10A—C10—H10B	109.5
O1—C1—C11	106.92 (19)	C1—C10—H10C	109.5
C2—C1—C11	111.2 (2)	H10A—C10—H10C	109.5
C10—C1—C11	111.3 (2)	H10B—C10—H10C	109.5
C3—C2—C1	122.2 (2)	C1—C11—H11A	109.5
C3—C2—H2A	118.9	C1—C11—H11B	109.5
C1—C2—H2A	118.9	H11A—C11—H11B	109.5
C2—C3—C4	119.3 (2)	C1—C11—H11C	109.5
C2—C3—H3	120.3	H11A—C11—H11C	109.5
C4—C3—H3	120.3	H11B—C11—H11C	109.5
C9—C4—C5	117.9 (2)	O3—C12—C6	119.8 (2)
C9—C4—C3	118.6 (2)	O3—C12—C13	116.2 (2)
C5—C4—C3	123.4 (2)	C6—C12—C13	124.0 (2)
O2—C5—C4	116.2 (2)	C12—C13—H13A	109.5
O2—C5—C6	121.7 (2)	C12—C13—H13B	109.5
C4—C5—C6	122.1 (2)	H13A—C13—H13B	109.5
C5—C6—C7	116.38 (19)	C12—C13—H13C	109.5
C5—C6—C12	118.6 (2)	H13A—C13—H13C	109.5
C7—C6—C12	125.0 (2)	H13B—C13—H13C	109.5
O4—C7—C8	121.91 (19)	O4—C14—H14A	109.5
O4—C7—C6	116.15 (18)	O4—C14—H14B	109.5
C8—C7—C6	121.93 (19)	H14A—C14—H14B	109.5
C7—C8—C9	119.2 (2)	O4—C14—H14C	109.5
C7—C8—H8	120.4	H14A—C14—H14C	109.5
C9—C8—H8	120.4	H14B—C14—H14C	109.5
			
C9—O1—C1—C2	−36.1 (3)	C5—C6—C7—O4	178.63 (19)
C9—O1—C1—C10	−157.02 (19)	C12—C6—C7—O4	−2.0 (3)
C9—O1—C1—C11	85.1 (2)	C5—C6—C7—C8	−0.1 (3)
O1—C1—C2—C3	25.1 (3)	C12—C6—C7—C8	179.2 (2)
C10—C1—C2—C3	141.0 (2)	O4—C7—C8—C9	−178.26 (18)
C11—C1—C2—C3	−93.6 (3)	C6—C7—C8—C9	0.4 (3)
C1—C2—C3—C4	−2.5 (4)	C1—O1—C9—C4	25.4 (3)
C2—C3—C4—C9	−11.4 (3)	C1—O1—C9—C8	−157.02 (19)
C2—C3—C4—C5	171.5 (2)	C5—C4—C9—O1	177.07 (19)
C9—C4—C5—O2	−179.63 (18)	C3—C4—C9—O1	−0.1 (3)
C3—C4—C5—O2	−2.6 (3)	C5—C4—C9—C8	−0.4 (3)
C9—C4—C5—C6	0.7 (3)	C3—C4—C9—C8	−177.6 (2)
C3—C4—C5—C6	177.72 (19)	C7—C8—C9—O1	−177.73 (18)
O2—C5—C6—C7	179.90 (18)	C7—C8—C9—C4	−0.2 (3)
C4—C5—C6—C7	−0.4 (3)	C5—C6—C12—O3	−1.2 (3)
O2—C5—C6—C12	0.5 (3)	C7—C6—C12—O3	179.5 (2)
C4—C5—C6—C12	−179.84 (19)	C5—C6—C12—C13	178.7 (2)
C14—O4—C7—C8	1.7 (3)	C7—C6—C12—C13	−0.6 (4)
C14—O4—C7—C6	−177.06 (19)		

### Hydrogen-bond geometry (Å, °)

**Table:**

*D*—H···*A*	*D*—H	H···*A*	*D*···*A*	*D*—H···*A*
O2—H2···O3	0.82	1.75	2.479 (2)	147
C8—H8···O3^i^	0.93	2.48	3.374 (3)	162

Symmetry codes: (i) *x*, *y*−1, *z*.
